# Retrograde Balloon Dilation outside the Main Branch Stent to Restore the Occlusion of Side Branch in Chronic Total Occlusion Bifurcation Lesions

**DOI:** 10.1155/2020/7625415

**Published:** 2020-01-11

**Authors:** Gao Hao-kao, Li Cheng-xiang

**Affiliations:** Department of Cardiology, The First Affiliated Hospital of Air Force Military Medical University, Xi'an 710032, China

## Abstract

Percutaneous coronary intervention (PCI) of a chronic total occlusion (CTO) can be challenging when a bifurcation is present at the distal cap. We described a case of retrograde balloon outside the main branch stent to restore the occlusion of side branch in CTO bifurcation lesion through the jailed wire.

## 1. Introduction

Percutaneous coronary intervention for chronic total occlusion (CTO-PCI) remains one of the most challenging clinical scenarios in interventional cardiology and a lower success rate compared to non-CTO lesions [1, 2]. Although the antegrade approach is a general approach for CTO recanalization, a retrograde approach is currently widely used in complex CTO lesion and improves the success rate when antegrade techniques are unlikely to succeed [3]. The presence of a bifurcation lesion in the context of a CTO represents an additional difficulty. The jailed balloon technique (JBT) is now emerging for protection of the side branch (SB) acquiring high procedural success rate without SB occlusion [4].

But in this case, we described a retrograde small profile balloon outside the main branch stent to restore the occlusion of posterolateral vessel (PLV) in right coronary artery (RCA) CTO bifurcation lesion via a jailed wire.

## 2. Case Report

A 63-year-old man with 40 years smoking habit and known coronary artery disease presented with Canadian Cardiovascular Society (CCS) class II angina. Two weeks before, the patient had just successfully undergone CTO-PCI of the left circumflex artery (LCx) in our hospital, and at this time, he was referred for PCI to CTO in the RCA. Twelve-lead electrocardiography showed slight ST-segment depression in II/III/avF leads. Ultrasound echocardiography revealed the preserved left ventricular ejection fraction (LVEF = 52%) and hypokinesia of the inferior and posterior left ventricular wall. Three days after admission, we performed PCI for RCA CTO.

**Figure 1:**
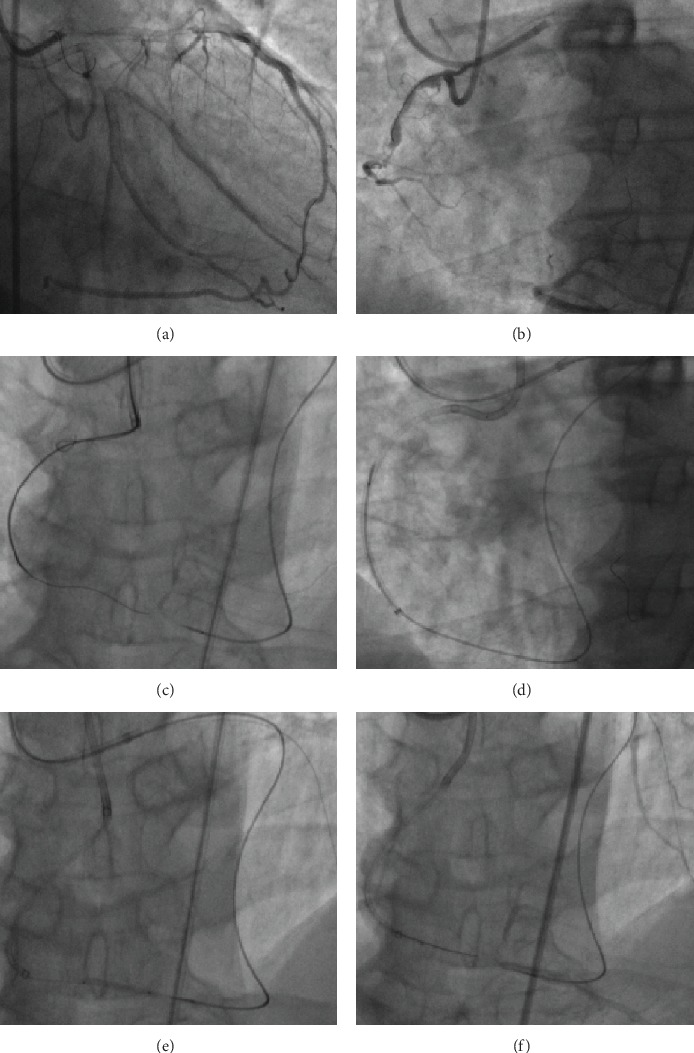
Recanalization of RCA CTO by a retrograde approach. (a) CAG showed LAD to RCA collaterals. (b) Simultaneous bilateral CAG showed long RCA CTO at bifurcation with large branches; (c) Corsair catheter in the right PDA placed retrograde via a epicardial collateral from the LAD, and tip injection showed antegrade wire in false lumen; (d) retrograde wire and Corsair into antegrade Guidezilla guide; (e) sequentially predilation RCA along antegrade RG3 wire; (f) retrograde angiography showed the severe disease in PLV.

This time, we performed simultaneous left and right coronary angiography (CAG). PCI was performed with right femoral and radial arteries route. The RCA was engaged with a 7F SAL1.0 guide in the radial artery and the left coronary artery (LCA) with a 7F EBU 3.75 guide in the radial artery. CAG demonstrated severe stenosis of the proximal RCA and CTO of the RCA extending from the RCA middle to the posterior descending artery (PDA) and PLV bifurcation (Figure 1(a)). The LCA had a severe stenotic lesion in the distal of LCx, and two stents (Abbott Vascular, USA) were implanted there. The RCA distal bifurcation was well filled by CC2 septal collateral channels and CC3 epicardial collaterals from the left anterior descending artery (LAD) (Figure 1(b)).

### 2.1. One Retrograde Approach Successfully Opened RCA-PD CTO

The entrance of CTO was stumpless especially with big side branch and longer CTO segment. The J-CTO score for RCA CTO was 2. The lesion was initially approached antegradely with a Fielder XT-R wire (Asahi Intecc, Japan) supported by a Corsair microcatheter (Asahi Intecc, Japan), the Fielder XT-R could not be advanced, and composite core Gaia third wire (Asahi Intecc, Japan) was subsequently used but also failed to reenter into distal true lumen which was confirmed in false lumen by contralateral angiography (Figure 1(c)). Therefore, we switched to retrograde approach. The LAD provided larger and less tortuous epicardial collaterals, and a SION guide wire (Asahi Intecc, Japan) with a Corsair microcatheter was introduced into the epicardial branch. Selective tip injection through the Corsair revealed the occlusion site at the bifurcation with large branches (Figure 1(c)). A Fielder XT-R guide wire was advanced “knuckled” up into the RCA CTO segment and overlaps with the antegrade wire at the middle part of CTO. The retrograde and antegrade guiding wires were difficult to kiss, and next, we used the reverse controlled antegrade and retrograde subintimal tracking (CART) technique to enlarge subintimal space with a 2.0 mm × 20 mm balloon. The reverse CART technique facilitated Guidezilla (Boston Scientific, Natick, MA, USA) into CTO middle segment for picking up retrograde wire and manipulating the retrograde Pilot 200 wire (Abbott Vascular, USA) and Corsair into a Guidezilla guide (Figure 1(d)). The Pilot 200 wire was then exchanged with a 330 cm RG3 wire (Asahi Intecc, Japan) that was externalized. After sequentially predilation with a 1.5 × 20 mm/2.0 × 20 mm balloon along an antegrade RG3 wire (Figure 1(e)), RCA antegrade flow was restored into the PDV, but retrograde angiography showed the severe disease in the PLV (Figure 1(f)).

### 2.2. Another Retrograde Approach to Save PLV flow outside the Main Vessel Stent

**Figure 2:**
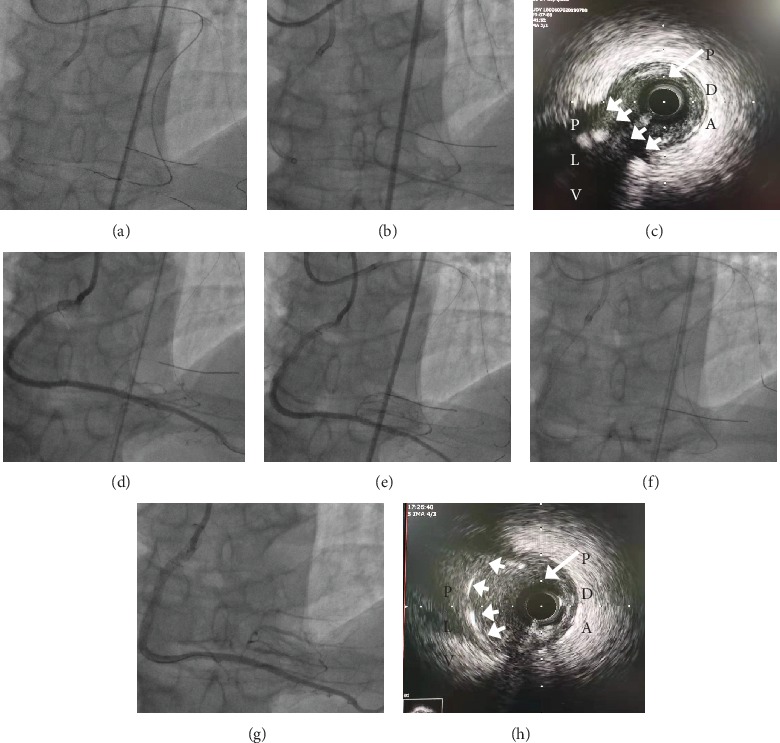
RCA bifurcation CTO PCI: (a) Corsair catheter in the right PLV placed retrograde via septal collaterals from the LAD and antegrade reverse bent wiring with the Crusade catheter failed into PLV; (b) recovery flow of PLV after retrograde balloon dilation; (c) IVUS showed PLV ostium had larger area before PDA stent; (d) angiography of RCA showing TIMI 1 grade flow in the right PLV after stent in the main vessel; (e) retrograde small profile balloon dilation to recovery PLV flow; (f) antegrade balloon and retrograde balloon outside the main branch stent final kissing technique; (g) the final result with flow in the right PDA and PLV; (h) IVUS showed the final PLV ostial area.

Several attempts to wire PLV in the antegrade direction using several guide wires failed, and even “reverse bent wiring with the Crusade catheter” for a Field XT-R wire recrossing to the PLV still failed because of the retroflexed angle (Figure 2(a)). To preserve the PDA/PLV bifurcation, a Sion retrograde wire with a 150 cm long Corsair microcatheter was advanced into PLV distal via the second septal vessel. A 1.5 × 20 mm balloon mini-Trek (Abbott Vascular, USA) successfully predilated the PLV for Thrombolysis In Myocardial Infarction (TIMI) 3 grade flow (Figure 2(b)). The intravascular ultrasound (IVUS) examination showed that the provisional stent from the PDV to the RCA proximal segment was appropriate with a low rate of PLV occlusion (Figure 2(c)). Stenting of the RCA into the PDV was performed using four Xience Prime stents (Abbott Vascular, USA). But angiography showed that PLV flow was slower in TIMI 1 grade flow after stents (Figure 2(d)). A retrograde 1.5 × 20 mm balloon successfully advanced through outside the PDA stent into the PLV to dilate it, and the flow was rescued (Figure 2(e)). To avoid the plaque shift to the PDA and preserve PLV flow, an antegrade 3.0 × 15 mm balloon Trek (Abbott Vascular, USA) with retrograde 2.0 × 20 mm Trek balloon performed the final kissing technique (FKT) to dilate bifurcation lesion with 14 atm, respectively (Figure 2(f)). Angiography and IVUS showed that PLV flow was successfully reserved (Figures 2(g) and 2(h)). At last, the retrograde guidewire and microcatheter were withdrawn, and RCA angiography showed no collateral circulation damage and rupture.

The total fluoroscopy time of the procedure was 170 minutes, and the total contrast volume delivered was 380 ml. The patient was followed up six months without symptom.

## 3. Discussion

In this case report, we described a new kissing technique for bifurcation lesion where one balloon was antegradely advanced in the main vessel and the other retrogradely advanced outside the main vessel stent. This was the reasonable approach because one of the limbs of the bifurcation could not be wired via the antegrade route and not rewired via retrograde approach. Al Aloul et al. [5] reported a case where a bifurcation lesion was treated with antegrade and retrograde “Head-to-Toe” kissing balloon angioplasty. But our case was different with his case for our retrograde balloon through outside of the main vessel stent for performing the kissing technique. To our knowledge, this was the first case report using this technique to dilate a bifurcation lesion.

Treating CTOs with a bifurcation at the distal cap can be challenging and often result in recanalization of only one of the branches [6]. Although new technique and devices have been developed, restoration of antegrade flow into both branches with large caliber still can be challenging. For this case, the CTO segment was very long with side branch. Antegrade approach might not be quite effective and failed to enter into the true lumen at bifurcation. Then, we turned to retrograde approach, in which epicardial collateral wire advanced into CTO segment and overlapped antegrade wire at the middle of CTO. We performed reverse CART technique and Guidezilla picking up retrograde wire with a Corsair guide, and RG3 wire externalization to completed RCA CTO.

The distal occlusion was located at the bifurcation site which had a markedly angulated PLV. It is considered that access to SB is usually more difficult in the case with the carina angle of more than 70 degrees and can be particularly difficult when the carina angle exceeds 90 degrees. Nomura et al. [7] reported the application of a “reverse bent wiring with the Crusade catheter” for wire crossing to a SB at the bifurcation lesion. It is actually a useful technique in our PCI center. But unfortunately, we failed into PLV by this technique in this case. Finally, we used a septal retrograde approach to preserve PLV flow by small profile balloon dilating it. Although IVUS examination showed the ostial area of PLV was enough with a low rate to occlude and also we conventionally adopted a jailed wire technique, the PLV still occlude after main branch stent. Kotsia et al. [8] reported the use of a retrograde Confianza Pro 12 guide wire rewiring into the main vessel stent through side branch and put stents followed by wire externalization. His technique might be safe and effective, but in our case, the carina angle of nearly 90 degrees in PLV made it more difficult to rewire though the ostial plaque of the PLV had been modified. The JBT is now emerging for protection of the side branch by reducing the risk of SB occlusion but with the risk of jailed balloon entrapment. In our case, once the retrograde balloon entrapment would cause severe injury. To overcome the dilemma situation, we tried small profile balloon (1.5 × 20 mm) advanced through the outside space of the main vessel stent and restored the flow of the PLV. Finally, the balloon kissing technique provided PL better flow without the main branch stent deformation.

Our case demonstrated an effective use of a retrograde approach for preserving a bifurcation at the distal CTO cap especially retrograde balloon dilating side branch outside of the main vessel stent. The retrograde balloon was smaller comparing with a traditional kissing technique in case of retrograde access injury, and the most important thing to remember was not to hard push the retrograde balloon. However, in similar cases, antegrade crossing was preferred if possible to keep side branch open, as it has a number of limitations. For example, it is difficult to cross or dilate small and tortuous septal channels and there exists the risk of septal injury. It is also easy to cause channel rupture, balloon trapping, and subintimal dissection.

However, given the technical difficulty and associated risks, the above technique of retrograde balloon dilating side branch outside of the main vessel stent should only be employed by experienced CTO operators. Additional cases are required to establish the success and reproducibility of this technique and to identify its limitations.

## 4. Conclusion

The technique of retrograde balloon dilating side branch outside the main vessel stent could be used to preserve both branches of a significant bifurcation at the CTO distal cap.
